# Short-term rehospitalization across the spectrum of age and insurance types in the United States

**DOI:** 10.1371/journal.pone.0180767

**Published:** 2017-07-10

**Authors:** Jordan B. Strom, Daniel B. Kramer, Yun Wang, Changyu Shen, Jason H. Wasfy, Bruce E. Landon, Elissa H. Wilker, Robert W. Yeh

**Affiliations:** 1 Richard A. and Susan F. Smith Center for Cardiovascular Outcomes Research, Division of Cardiovascular Medicine, Beth Israel Deaconess Medical Center, Boston, Massachusetts, United States of America; 2 Department of Biostatistics, Harvard T.H. Chan School of Public Health, Boston, Massachusetts, United States of America; 3 Division of Cardiovascular Medicine, Massachusetts General Hospital, Boston, Massachusetts, United States of America; 4 Department of Healthcare Policy, Harvard Medical School, Boston, Massachusetts, United States of America; University of Utah School of Medicine, UNITED STATES

## Abstract

Few studies have examined rates and causes of short-term readmissions among adults across age and insurance types. We compared rates, characteristics, and costs of 30-day readmission after all-cause hospitalizations across insurance types in the US. We retrospectively evaluated alive patients ≥18 years old, discharged for any cause, 1/1/13-11/31/13, 2006 non-federal hospitals in 21 states in the Nationwide Readmissions Database. The primary stratification variable of interest was primary insurance. Comorbid conditions were assessed based on Elixhauser comorbidities, as defined by administrative billing codes. Additional measures included diagnoses for index hospitalizations leading to rehospitalization. Hierarchical multivariable logistic regression models, with hospital site as a random effect, were used to calculate the adjusted odds of 30-day readmissions by age group and insurance categories. Cost and discharge estimates were weighted per NRD procedures to reflect a nationally representative sample. Diagnoses for index hospitalizations leading to rehospitalization were determined. Among 12,533,551 discharges, 1,818,093 (14.5%) resulted in readmission within 30 days. Medicaid insurance was associated with the highest adjusted odds ratio (AOR) for readmission both in those ≥65 years old (AOR 1.12, 95%CI 1.10–1.14; p <0.001), and 45–64 (AOR 1.67, 95% CI 1.66–1.69; p < 0.001), and Medicare in the 18–44 group (Medicare vs. private insurance: AOR 1.99, 95% CI 1.96–2.01; p <0.001). Discharges for psychiatric or substance abuse disorders, septicemia, and heart failure accounted for the largest numbers of readmissions, with readmission rates of 24.0%, 17.9%, 22.9% respectively. Total costs for readmissions were 50.7 billion USD, highest for Medicare (29.6 billion USD), with non-Medicare costs exceeding 21 billion USD. While Medicare readmissions account for more than half of the total burden of readmissions, costs of non-Medicare readmissions are nonetheless substantial. Medicaid patients have the highest odds of readmission in individuals older than age 44, commonly due to hospitalizations for psychiatric illness and substance abuse disorders. Medicaid patients represent a population at uniquely high risk for readmission.

## Introduction

Unplanned readmissions after hospital discharge are common, costly, and an important contributor to health care utilization. More than half of Medicare beneficiaries are readmitted within one year of discharge, accounting for billions of expenditures annually beyond those associated with the index admissions [1–3]. Accordingly, reducing hospital readmissions has become a national health care priority. The Patient Protection and Affordable Care Act [4] authorized the Department of Health and Human Services to establish a Hospital Readmissions Reduction Program, which utilizes hospital-based payment incentives to curb readmissions. Subsequently, the Centers for Medicare and Medicaid Services (CMS) implemented mandatory public reporting of and conditional reimbursement for 30-day risk standardized readmission measures for five common medical conditions or surgical procedures [5].

Despite the heightened attention towards reducing readmissions, only limited research has assessed patient and hospital characteristics associated with hospital readmissions among patients spanning the full range of age and insurance categories. In particular, the burden of readmissions among younger patients, and those not insured by Medicare are less well characterized. Thus, the goals of this study were to assess the rates, characteristics, and costs of hospital readmissions across all age and insurance categories, and to identify factors associated with all-cause readmissions.

## Materials and methods

### Data sources

The study examined admissions within the 2013 Nationwide Readmissions Database (NRD), an all-payer claims database that includes information on non-federal hospital claims from 21 geographically disparate states, with weighted estimates accounting for 36 million discharges in the United States. After exclusion of rehabilitation and long-term acute care hospitals, discharges with missing or questionable patient linkage numbers (more than 20 discharges per year, hospitalized after discharged dead, or overlapping inpatient stays), or discharges from hospitals with more than 50% of total discharges excluded for any of the above reasons, the NRD represents an 85% sample of all discharges in the 2013 State Inpatient Databases (SIDs) as part of the Healthcare Costs and Utilization Project (HCUP), a joint federal, state, and industry partnership sponsored by the Agency for Healthcare Research and Quality [6]. The 2013 NRD was created using verified patient linkage identification numbers that allow tracking of hospitalizations for a given patient across a state and allows calculation of all-cause readmission rates across all-payers and for the uninsured. The NRD and affiliated databases have been previously used to examine national rates of hospital readmissions [7]. The validity and internal consistency of data elements are subject to quality review by an independent contractor [8]. Discharge data from the NRD encompasses 49.1 percent of all United States hospitalizations (HCUP), and was available only for the year 2013 at the time of analysis [6]. Discharges with missing or invalid patient linkage identifiers are excluded from the NRD in the analytical dataset [8].

This study was determined to be exempt from review by the Beth Israel Deaconess Medical Center Institutional Review Board and is consistent with the HCUP Data Use Agreement.

### Study population

Discharges of patients aged ≥18 years old hospitalized for any condition who survived to discharge were eligible for inclusion. Admissions that included transfers between acute care facilities were merged to form single episodes of care [9]. Additionally, in order to restrict the analysis to unscheduled hospitalizations, ICD-9-CM V codes were excluded as they mainly represented elective or scheduled hospitalizations [10].

### Variables

Clinical and comorbidity variables were determined by the International Classification of Diseases, Clinical Modification, Version 9 (ICD-9-CM) codes included in the HCUP Elixhauser Comorbidity Software, Version 3.7 (Table A in **S1 File**) [11]. Demographic variables identified included sex, age, and insurance status (Medicare, Medicaid, private insurance, self-pay, unknown). Insurance status was defined by the primary payer listed on the index hospital discharge. Information on secondary payers is not included in the NRD [6]. Patients 65 and older whose primary insurance was Medicaid were assumed to be eligible to enroll in Medicare (“dual eligible”). Among Medicare-insured patients, fee-for-service and Medicare Advantage beneficiaries are included in the NRD under a single category.

Clinical variables ascertained included diagnosis of heart failure, myocardial infarction, valvular disease, hypertension, peripheral vascular disease, hypothyroidism, diabetes mellitus, chronic pulmonary disease, pulmonary circulatory disorders, stroke, dementia, paralysis, alcohol or drug abuse, psychosis, depression, coagulopathy, metastatic cancer, lymphoma, weight loss, liver disease, renal failure, any chronic condition, major procedure during index hospitalization, and source of admission, based on billing codes as previously described **(Table A in S1 File**) [11]. Index admissions were classified according to discharge diagnosis. Missing insurance status was categorized as “unknown” in the analysis, and was limited to 4.8% of the overall sample. Data on race [6], secondary payer data, and an individual’s household income, were missing and excluded in the final model. No other variables included in the model had missing data and thus no imputation was performed. Additionally, results are adjusted for the median household income of an individual’s zip code which has previously been correlated to readmission risk [12–13].

### Outcomes

The primary outcome of interest in the study was 30-day all-cause readmission, defined as hospitalizations occurring after one day and within one month of discharge from an index hospitalization occurring from January 1, 2013 to November 31, 2013, as described previously [9]. Hospitalizations through November were chosen to allow for a one month follow-up interval to assess 30-day readmissions [9]. Only the first readmission within 30 days of the index hospitalization was assessed, similar to methods used by CMS in public reporting [14]. Thereafter, subsequent rehospitalization was considered to be a new index admission. As the unit of analysis was hospitalizations, a given patient could be included more than once if they were subsequently readmitted. We calculated a mean annual rate of 1.1 rehospitalizations per patient in the NRD, and thus no adjustment was made for clustering of rehospitalizations at a patient level as it was unlikely to change effect estimates significantly. Total weighted costs in 2013 United States dollars (USD) were determined for 30-day readmissions and stratified by insurance and age categories using the HCUP Cost-to-charge ratio [15]. The HCUP Cost-to-charge ratio uses estimates of all-payer inpatient costs from the CMS, is internally validated by HCUP, and is not subject to discounting [16].

### Statistical analysis

Baseline characteristics of patients were examined at the time of index hospitalization among patients readmitted within 30 days and those not readmitted in aggregate and stratified by insurance type. Categorical data are expressed as frequencies and percentages and continuous data as means and standard deviations. Aggregate rates of readmissions were determined and stratified by age group (18–44, 45–65, and ≥ 65 years old) and insurance category, and separately for each of the most common discharge diagnoses leading to subsequent 30-day rehospitalization.

To determine the relationship between age and insurance status to 30-day readmission rates, separate hierarchical logistic regression models were built with insurance status as primary predictor, for each age category, and adjusted for all patient level covariates, as determined by Elixhauser diagnoses on discharge. Hospital site was included as a random effect in the mixed model. Mixed models were used in order to account for clustering of patient-level outcomes by hospital in order to obtain accurate standard errors estimates and efficient statistical inferences. Models were adjusted for clinical comorbidities as determined by the method of Elixhauser, without subsequent variable selection. Dummy variables were created for non-binary categorical covariates and reference groups were chosen as the largest sample in each age category. Because the NRD is sampled in a manner that is intended to reflect a nationally representative population, total cost and discharge estimates were weighted according to HCUP recommendations, as previously described [17]. Adjusted odds ratios and 95% confidence intervals (CI) were generated from these analyses. 95% confidence intervals for proportions were computed using normal approximation All analyses were conducted with SAS version 9.4 (SAS Institute Inc., Cary, NC). P-values were two sided and an alpha level of 0.05 was considered statistically significant.

## Results

### Patient and hospital characteristics

A total of 12,533,551 discharges from 2,006 hospitals were included in the analysis [18]. Of these discharges, 1,818,093 (14.5%) were followed by readmission within 30 days of the index hospitalization. Readmitted patients had a mean age of 60.4±19, were 52.3% female, and 95.7% had at least one chronic condition (**Table 1**).

**Table 1:** Baseline characteristics among readmitted and non-readmitted patients by age category^a^.

	Total		Age					
			18–44, y		45–64, y		65+, y	
								
Variable Name	Readmitted (N = 1 818 093)	Not readmitted (N = 10 715 458)	Readmitted (N = 393 191)	Not Readmitted (N = 3 371 155)	Readmitted (N = 590 885)	Not readmitted (N = 3 067 274)	Readmitted (N = 834 017)	Not Readmitted (N = 4 227 029)
**Age, mean (SD), y**	60.4 (19.1)	56.3 (20.8)	32.2 (7.5)	30.7 (7.2)	55.2 (5.6)	55.2 (5.6)	79.0 (2.6)	78.9 (2.6)
**Female**	950666 (52.3)	6504940 (60.7)	233396 (59.4)	2579990 (76.5)	273178 (46.2)	273178 (46.2)	444092 (53.2)	1481304 (35.0)
**History of Heart Failure**	346486 (19.1)	1203372 (11.2)	14511 (3.7)	41601 (1.2)	88176 (14.9)	275799 (9.0)	243799 (29.2)	885972 (21.0)
**History of Acute Myocardial Infarction**	23206 (1.3)	86383 (0.8)	719 (0.2)	2965 (0.1)	5853 (1.0)	21722 (0.7)	16634 (2.0)	61696 (1.5)
**Hypothyroidism**	221583 (12.2)	1159687 (10.8)	17930 (4.6)	119331 (3.5)	57364 (9.7)	295271 (9.6)	146289 (17.5)	745085 (17.6)
**Other Neurologic Disease**	166046 (9.1)	722003 (6.7)	26406 (6.7)	100732 (3.0)	50779 (8.6)	196512 (6.4)	88861 (10.7)	424759 (10.0)
**Valvular Disease**	77610 (4.3)	342413 (3.2)	4249 (1.1)	19204 (0.6)	14533 (2.5)	61594 (2.0)	58828 (7.1)	261615 (6.2)
**Hypertension**	1001183 (55.1)	5058609 (47.2)	86431 (22.0)	380769 (11.3)	329103 (55.7)	1649495 (53.8)	585649 (70.2)	3028345 (71.6)
**History of Stroke**	28425 (1.6)	136452 (1.3)	632 (0.2)	3171 (0.1)	5802 (1.0)	27731 (0.9)	21991 (2.6)	105550 (2.5)
**Coagulopathy**	116577 (6.4)	458340 (4.3)	15116 (3.8)	81029 (2.4)	44163 (7.5)	149826 (4.9)	57298 (6.9)	227485 (5.4)
**Renal failure**	338792 (18.6)	1122814 (10.5)	25360 (6.4)	56865 (1.7)	94862 (16.1)	268169 (8.7)	218570 (26.2)	797780 (18.9)
**Chronic pulmonary disease**	411640 (22.6)	1787013 (16.7)	46464 (11.8)	254346 (7.5)	136866 (23.2)	570192 (18.6)	228310 (27.4)	962475 (22.8)
**Pulmonary circulatory disorders**	52631 (2.9)	189166 (1.8)	4545 (1.2)	14014 (0.4)	14310 (2.4)	46704 (1.5)	33776 (4.0)	128448 (3.0)
**Dementia**	13251 (0.7)	61429 (0.6)	33 (0.0)	103 (0.0)	1201 (0.2)	4019 (0.1)	12017 (1.4)	57307 (1.4)
**Alcohol or Drug Abuse**	205963 (11.3)	842286 (7.9)	75367 (19.2)	336385 (10.0)	103197 (17.5)	385645 (12.6)	27399 (3.3)	120256 (2.8)
**Peripheral Vascular Disease**	145290 (8.0)	571459 (5.3)	3916 (1.0)	12258 (0.4)	36102 (6.1)	130187 (4.2)	105272 (12.6)	429014 (10.1)
**Metastatic Cancer**	61676 (3.4)	195902 (1.8)	4806 (1.2)	12327 (0.4)	25220 (4.3)	73063 (2.4)	31650 (3.8)	110512 (2.6)
**Major procedure**	361847 (19.9)	3435596 (32.1)	67578 (17.2)	1078327 (32.0)	123135 (20.8)	1106734 (36.1)	171134 (20.5)	1250535 (29.6)
**Psychosis**	118733 (6.5)	475282 (4.4)	31012 (7.9)	130252 (3.9)	52953 (9.0)	195538 (6.4)	34768 (4.2)	149492 (3.5)
**Liver disease**	85339 (4.7)	300811 (2.8)	13034 (3.3)	51318 (1.5)	48846 (8.3)	164092 (5.3)	23459 (2.8)	85401 (2.0)
**Depression**	217345 (12.0)	1067778 (10.0)	41205 (10.5)	206120 (6.1)	83326 (14.1)	400612 (13.1)	92814 (11.1)	461046 (10.9)
**Diabetes Mellitus**	535823 (29.5)	2320300 (21.7)	45357 (11.5)	179370 (5.3)	190926 (32.3)	814275 (26.5)	299540 (35.9)	1326655 (31.4)
**Lymphoma**	22150 (1.2)	72943 (0.7)	1733 (0.4)	5189 (0.2)	6508 (1.1)	20069 (0.7)	13909 (1.7)	47685 (1.1)
**Paralysis**	50860 (2.8)	207446 (1.9)	8605 (2.2)	34240 (1.0)	17590 (3.0)	68934 (2.2)	24665 (3.0)	104272 (2.5)
**Weight Loss**	123459 (6.8)	422739 (3.9)	13769 (3.5)	43781 (1.3)	39219 (6.6)	120249 (3.9)	70471 (8.4)	258709 (6.1)
**Any Chronic Condition**	1739893 (95.7)	9285190 (86.7)	330369 (84.0)	2061583 (61.2)	580095 (98.2)	2977234 (97.1)	829429 (99.4)	4246373 (100.0)
**Admitted from Emergency Department**	1346300 (74.1)	6300096 (58.8)	260212 (66.2)	1358492 (40.3)	441121 (74.7)	1952554 (63.7)	644967 (77.3)	2989050 (70.7)
**Elective Admission**	248737 (13.7)	2715576 (25.3)	60379 (15.4)	1069400 (31.7)	80905 (13.7)	776147 (25.3)	107453 (12.9)	870029 (20.6)
**In-state resident**	1759254 (96.8)	10259042 (95.7)	379684 (96.6)	3256070 (96.6)	571339 (96.7)	2920676 (95.2)	808231 (96.9)	4082296 (96.6)
**Insurance**								
**Medicare**	1009941 (55.5)	4758913 (44.4)	58198 (14.8)	185080 (5.5)	189732 (32.1)	710594 (23.2)	762011 (91.4)	3109170 (73.6)
**Medicaid**	312019 (17.2)	1775009 (16.6)	147526 (37.5)	1172608 (34.8)	149891 (25.4)	537878 (17.5)	14602 (1.8)	57295 (1.4)
**Self-Pay**	87464 (4.8)	536813 (5.0)	45216 (11.5)	278943 (8.3)	39736 (6.7)	244394 (8.0)	2512 (0.3)	13476 (0.3)
**Private**	332589 (18.3)	3119955 (29.1)	114103 (29.0)	1515916 (45.0)	173041 (29.3)	1323934 (43.2)	45445 (5.4)	45445 (6.6)
**Unknown**	76080 (4.2)	524768 (4.9)	28148 (7.2)	218608 (6.4)	38485 (6.5)	250474 (8.1)	9447 (1.1)	1001543 (18.1)

^a^All p-values for comparisons are < 0.001 and two-tailed. Categorical variables are expressed as counts and percentages and continuous variables as means and standard deviations.

Abbreviations: y = years. SD = standard deviation.

### Readmission rates by insurance type

**Fig 1:**
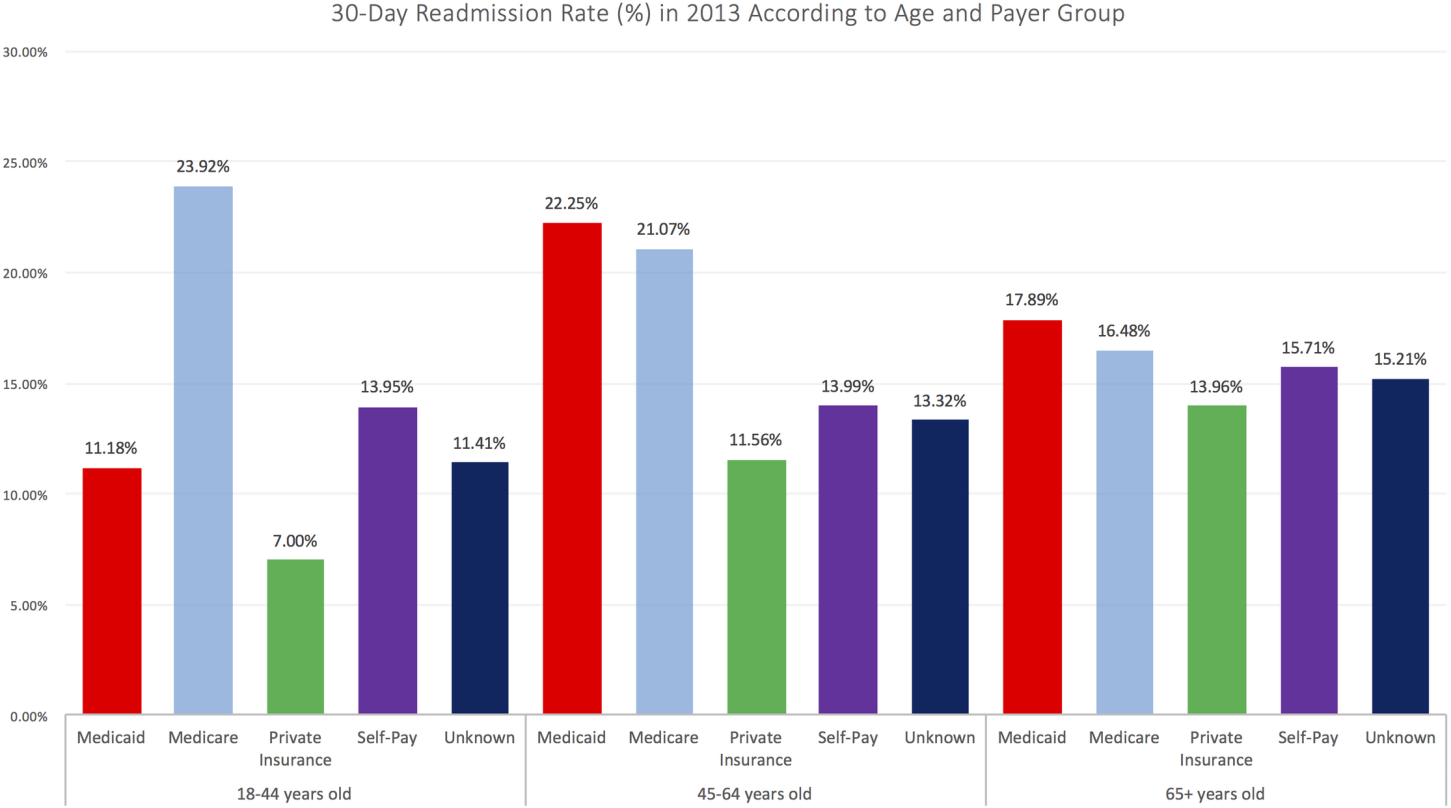
Readmission rates stratified by age and insurance category*. *All rates are expressed as percentages by age category.

Readmissions among Medicare-insured patients accounted for 56%, 95% CI 56.0–56.0% of all 30-day readmissions, followed by Private insurance (18.3%, 95% CI 18.3–18.3%), Medicaid (17.2%, 95% CI 17.2–17.2%), and self-pay (4.8%, 95% CI 4.8–4.8%). Unadjusted rates of 30-day readmission were highest in Medicare (17.5%, 95% CI 17.5–17.5%) followed by Medicaid (15.0%, 95% CI 15.0–15.0%), self-pay (14.0%, 95% CI 14.0–14.0%), and private insurance (9.6%, 95% CI 9.6–9.6%). Among patients age 65 and older, 30-day readmission rates were highest in Medicaid-insured population (17.9%, 95% CI 17.9–17.9%), followed by Medicare (16.5%, 95% CI 16.5–16.5%), self-pay (15.7%, 95% CI 15.7–15.7%), unknown (15.2%, 95% CI 15.2–15.2%), and private insurance (14.0%, 95% CI 14.0–14.0%). Among those 45–65 year old, this relationship was similar, with rates of 30-day readmission being highest in Medicaid (22.3%, 95% CI 22.3–22.3%), followed by Medicare (21.1%, 95% CI 21.1–21.1%), self-pay (14.0%, 95% CI 14.0–14.0%), unknown (13.3%, 95% CI 13.3–13.3%), and private insurance (11.6%, 95% CI 11.6–11.6%). Among patients 18–44 years old, rates of 30-day readmission were highest in Medicare (23.9%, 95% CI 23.9–23.9%), followed by self-pay (14.0%, 95% CI 14.0–14.0%), unknown (11.4%, 95% CI 11.4–11.4%), Medicaid (11.2%, 95% CI 11.2–11.2%), and private insurance (7.0%, 95% CI 7.0–7.0%). (**Fig 1)**.

After adjustment for sex, comorbidities, and admission source, Medicaid was associated with the highest risk of 30-day readmission in patients older than age 65 (AOR 1.12 vs. Medicare, 95% CI 1.10–1.14; p < 0.05) as well as in patients age 45–64 (AOR 1.67 vs. private insurance, 95% CI 1.66–1.69; p<0.05) (**Table 2**). Among patients age 18–44, Medicare was associated with the highest adjusted odds of readmissions (AOR 1.99 vs. private insurance, 95% CI 1.96–2.01; p <0.05), followed by Medicaid (AOR 1.52, 95% CI 1.5–1.53; p <0.05), and self-pay (AOR 1.39, 95%CI 1.37–1.41; p <0.05).

**Table 2:** Adjusted odds of 30-day readmission by age category and payer group^a^.

	Age Group		
Payer	18–44	45–64	65+
**Self-Pay**	1.39 (1.37–1.41)	1.07 (1.06–1.08)	0.96 (0.92–1.00)
**Medicaid**	1.52 (1.52–1.53)	1.67 (1.66–1.69)	1.12 (1.10–1.14)
**Medicare**	1.99 (1.96–2.01)	1.56 (1.55–1.57)	Ref
**Private Insurance**	Ref	Ref	0.87 (0.86–0.88)

^a^Effect estimates are expressed as adjusted odds ratios and 95% confidence intervals by age and payer category.

Covariates included in **Table A in S1 File**.

### Costs of readmissions

Total weighted costs for 30-day readmissions in 2013 were 50.7 billion USD, of which 58% (29.6 billion USD) were for Medicare patients and 42% (21.1 billion USD) were for non-Medicare patients (private insurance [20%], Medicaid [15%], unknown insurance [4%], and self-pay [3%]). Costs were highest for Medicaid in the 18–44 year old category (3.1 billion USD), private insurance in the 45–64 year old category (5.8 billion USD), and Medicare in the 65 and older category (22.3 billion USD).

### Discharge diagnoses associated with readmission

The most common discharge diagnoses for index hospitalizations resulting in readmission (**Table B in S1 File**) were septicemia (4.7% of total readmissions), heart failure (4.6%), cellulitis (3.1%), chronic bronchitis (2.8%), and cardiac dysrhythmias (2.7%). The frequency of these conditions differed markedly by age group and insurance type **(Table 3)**. Discharge diagnoses associated with readmission were more closely related to age group than insurance type. (**Table C in S1 File**) Among younger adults ages 18–44 years old, psychiatric disease, complications of pregnancy, and diabetes were the most common primary discharge diagnoses that were followed by readmission. Among adults 45–64 years old, discharges for septicemia, heart failure, and psychiatric disease were most common, the latter primarily in the publicly insured subgroups. In patients older than 65, heart failure, septicemia, cardiac dysrhythmias, and chronic bronchitis were the most common diagnoses leading to readmission.

**Table 3:** Most common discharge diagnoses for the index admission for patients readmitted within 30 days stratified by payer and rates of readmission.

**Medicaid**					**Medicare**				
**Rank**	**Diagnosis**	**ICD-9-CM Code**	**Number of All-Cause 30-Day Readmissions**	**30-Day Readmission Rate (%)**	**Rank**	**Diagnosis**	**ICD-9-CM Code**	**Number of All Cause 30-Day Readmissions**	**30-Day Readmission Rate (%)**
1	Schizophrenic Disorders	295.xx	14,574	25.67	1	Heart Failure	428.xx	63,814	23.04
2	Episodic Mood Disorders	296.xx	13,353	19.86	2	Septicemia	038.xx	60,117	19.02
3	Diabetes Mellitus	250.xx	11,964	22.74	3	Chronic bronchitis	491.xx	36,916	20.64
4	Septicemia	038.xx	10,498	20.57	4	Cardiac dysrhythmias	427.xx	35,526	17.38
5	Heart Failure	428.xx	9,368	28.64	5	Pneumonia	486.xx	33,630	16.73
**Private Insurance**		** **	** **	** **	**Self-Pay**				
**Rank**	**Diagnosis**	**ICD-9-CM Code**	**Number of All Cause 30-Day Readmissions**	**30-Day Readmission Rate (%)**	**Rank**	**Diagnosis**	**ICD-9-CM Code**	**Number of All Cause 30-Day Readmissions**	**30-Day Readmission Rate (%)**
1	Septicemia	038.xx	10,560	14.21	1	Diabetes Mellitus	250.xx	3,967	17.04
2	Cardiac dysrhythmias	427.xx	7,817	13.08	2	Episodic Mood Disorders	296.xx	3,925	14.89
3	Episodic Mood Disorders	296.xx	7,767	11.81	3	Respiratory or Chest Symptoms	786.xx	3,634	21.44
4	Respiratory or Chest Symptoms	786.xx	7,273	15.9	4	Diseases of Pancreas	577.xx	2,510	16.08
5	Diabetes Mellitus	250.xx	6,914	15.74	5	Alcohol-induced Mental Disorder	291.xx	2,471	16.85

## Discussion

While prior examinations of short-term hospital readmissions have focused primarily on the Medicare population, this study identified the considerable number and cost of non-Medicare readmissions, as well as the high burden of readmission among patients admitted for psychiatric disease and substance abuse disorders. Non-Medicare readmissions accounted for 44% of all readmissions in the US and more than 20 billion USD in direct costs in 2013. In examining relative rates of readmissions across all age and insurance categories, we found that Medicaid-insured patients accounted for high rates of readmission in all but the youngest age group. In the non-elderly group, and particularly in the young Medicare and Medicaid populations, psychiatric disease and substance abuse were the most important contributors to rehospitalization, representing a tenth of all discharges leading to readmission, and almost one fifth of discharges leading to readmission in the Medicare and Medicaid insured population. As individuals may become eligible for Medicare after two years of disability payments [19], the young Medicare group additionally represents a distinct group at uniquely high risk of readmission, as shown by Medicare patients having the highest rate of readmission in the youngest age group.

While the role of mental and behavioral health in Medicaid readmissions has previously been described [20], our study suggests that this role is not unique to Medicaid and is more closely related to age group than insurance category. In our study, 37.5% of those age 18–44 and 40.5% of those age 45–64 who were readmitted had a history of psychiatric disease or substance abuse compared with 18.6% in those over age 65. Given rising morbidity and mortality attributable to suicides, alcohol, and drug poisonings [21], the role of psychiatric disease and substance abuse in non-elderly readmissions may reflect national disease trends. Nevertheless, our study suggests the importance of developing and implementing interventions known to reduce psychiatric readmissions in the young population, regardless of insurance status [22]. Given that more than half of all readmissions followed a hospitalization for one of 33 conditions, there is tremendous potential to reduce readmissions by focusing funding and efforts on selected conditions. Furthermore, given the importance of readmissions to risk contracts in accountable care organizations (ACOs) [23], the importance of psychiatric disease in Medicaid readmissions in this study bolsters efforts to intensify resources for psychiatric disease in the development of Medicaid ACOs [24]. While we have shown that insurance status is related to readmission risk and the type of readmission, it is unknown whether changing insurance would result in a lower risk of readmission and the analysis was not intended to suggest a causal mechanism. Rather, as prior literature has shown some success at reducing readmissions through multiple targeted interventions [25], identifying high risk-groups may allow focusing of such resources on cohorts most likely to be readmitted.

Medicaid beneficiaries over the age of 65 –patients likely to be eligible to receive concomitant Medicare benefits (i.e. “dual eligibles”)—had the highest risk of readmission among all patients even after adjustment for patient comorbidities accounting for hospital-based clustering. As such, our study suggests that the uniquely high risk of this group of patients, which may be related to factors outside the sphere of influence of hospitals, should be accounted for in structuring hospital based performance measures and policy planning [13, 26–32]. As Medicare does not currently adjust for socioeconomic status in reporting of risk adjusted readmissions [13, 26–32], the uniquely high risk of readmissions among potential dual-eligible patients has implications for safety net hospitals who more commonly experience penalties for excess readmissions [28]. It should be noted, however, that not all dual-eligible patients would have been included in the elderly Medicaid-insured group in this analysis, as only information for the primary payer is available in the NRD. Thus, dual-eligible patients for whom Medicare was the primary payer would have been included in the elderly Medicare group in this study.

The current study has several limitations worth noting. First, the study is retrospective and included a limited number of variables, and therefore is subject to residual confounding and may differ from the true causal effect. Second, while many claims have been validated [33], there may be inaccuracies in coding that may introduce imprecision in our estimates. Third, the analysis was limited to studying 30-day readmissions and it is plausible that patterns in readmissions after 30 days may be systematically different than those within 30 days, though studies in the Medicare population have suggested that readmission risk persists over time [1] without an effect of time on diagnoses associated with readmission [34]. Fourth, in-hospital mortality is included as a discharge in the NRD as these are possible readmission records and thus estimates may underestimate the overall probability of readmission [17]. Fifth, as only primary insurance was available for analysis, Medicaid beneficiaries over age 65, while likely eligible to receive Medicare, may not be concomitantly enrolled in Medicare, and furthermore, many dual eligible individuals may have Medicare as their primary insurance. Thus, conclusions regarding dual-eligible individuals may not represent the overall dual-eligible population. Sixth, given the absence of important confounders such as race and an individual’s income, causality cannot be inferred and the analysis was intended to be predictive. Seventh, although the NRD is the largest all payer national database in the US for readmissions, nevertheless, it may not be completely representative of national trends. Finally, ICD-9-CM V codes, which represent occasions for which settings other than disease or injury results in a clinical encounter or influences care, were excluded from the analysis given the frequency to which they were used for elective readmissions, but it is possible that some may represent unscheduled rehospitalizations, and thus readmission estimates may under-represent the overall percentage of readmissions.

## Conclusions

While Medicare readmissions account for more than half of the total burden of readmissions, non-Medicare readmissions are frequent and are associated with substantial cost. Psychiatric disease, substance abuse, and chronic illness are frequently associated with rehospitalization in the non-Medicare population.

## Supporting information

S1 File Table A. Comorbid Conditions Included in the Agency for Healthcare Research and Quality Risk Adjustment Model.
Table B. Most Common Discharge Diagnoses Leading to Readmission Overall.
Table C. Discharge Diagnoses of Index Hospitalizations, Numbers, and Rates of Thirty Day Rehospitalizations.
(PDF)
