# Supplementary material for: Short-term rehospitalization across the spectrum of age and insurance types in the United States
Source: PLoS One. 2017 Jul 10;12(7):e0180767. doi: 10.1371/journal.pone.0180767 (PMC5507267; doi:10.1371/journal.pone.0180767)
Supplement: S1 File — Table A. Comorbid Conditions Included in the Agency for Healthcare Research and Quality Risk Adjustment Model. Table B. Most Common Discharge Diagnoses Leading to Readmission Overall. Table C. Discharge Diagnoses of Index Hospitalizations, Numbers, and Rates of Thirty Day Rehospitalizations. (PDF) [file pone.0180767.s001.pdf]

SUPPORTING INFORMATION

S1 Table A. Comorbid Conditions Included in the Agency for Healthcare Research and Quality Risk Adjustment Model

Agency for Healthcare Research and Quality Risk Adjustment Model Variables	ICD-9CM Diagnosis Codes
Congestive heart failure	398.91, 402.01, 402.11, 402.91, 404.01, 404.03, 404.11, 404.13, 404.91, 404.93, 428.0- 428.9
Valvular disease	093.20- 093.24, 394.0- 397.1, 397.9, 424.0- 424.99, 746.3- 746.6, V42.2, V43.3
Pulmonary circulation disorders	415.11- 415.19, 416.0- 416.9, 417.9
Peripheral vascular disorders	440-440.9, 441.00- 441.9, 442.0- 442.9, 443.1- 443.9, 444.21- 444.22, 447.1,449, 557.1, 557.9, V43.4
Paralysis	342.0- 344.9, 438.20- 438.53, 780.72
Other neurological disorders	330.1- 331.9, 332.0, 333.4, 333.5, 333.71, 333.72, 333.79, 333.85, 333.94, 334.0- 335.9, 338.0, 340, 341.1- 341.9, 345.00- 345.11, 345.2- 345.3, 345.40- 345.91, 347.00- 347.01, 347.10- 347.11, 649.40- 649.44, 768.7, 768.70, 768.71, 768.72, 780.3, 780.31, 780.32, 780.33, 780.39, 780.97, 784.3
Chronic pulmonary disease	490-492.8, 493.00- 493.92, 494- 494.1, 495.0-505, 506.4
Diabetes, uncomplicated	249.00- 249.31, 250.00- 250.33, 648.00- 648.04
Diabetes with chronic complications	249.40- 249.91, 250.40- 250.93, 775.1
Hypothyroidism	243-244.2, 244.8, 244.9

Renal failure	403.01, 403.11, 403.90, 403.91, 404.02, 404.03, 404.12, 404.13, 404.92, 404.93, 585.3, 585.4, 585.5, 585.6, 585.9, 586, V42.0, V45.1, V45.11, V45.12, V56.0-V56.32, V56.8
Liver disease	070.22, 070.23, 070.32, 070.33 , 070.44, 070.54, 456.0, 456.1, 456.20, 456.21, 571.0, 571.2, 571.3, 571.40- 571.49, 571.5, 571.6, 571.8, 571.9, 572.3, 572.8, 573.5, V42.7
Peptic ulcer disease excluding bleeding	531.41, 531.51, 531.61, 531.70, 531.71, 531.91, 532.41, 532.51, 532.61, 532.70, 532.71, 532.91, 533.41, 533.51, 533.61, 533.70, 533.71, 533.91, 534.41, 534.51, 534.61, 534.70, 534.71, 534.91
Acquired immune deficiency syndrome	042-044.9
Lymphoma	200.00- 202.38, 202.50- 203.01, 203.02- 203.82, 203.8- 203.81, 238.6, 273.3
Metastatic cancer	196.0- 199.1, 209.70, 209.71, 209.72, 209.73, 209.74, 209.75, 209.79, 789.51
Solid tumor without metastasis	140.0- 172.9, 174.0- 175.9, 179-195.8, 209.00- 209.24, 209.25-209.3, 209.30- 209.36, 258.01-258.03
Rheumatoid arthritis/collagen vascular diseases	701.0, 710.0- 710.9, 714.0-714.9, 720.0- 720.9, 725
Coagulopathy	286.0- 286.9, 287.1, 287.3-287.5, 289.84, 649.30- 649.34
Obesity	278.0, 278.00, 278.01, 278.03, 649.10- 649.14, 793.91, V85.30-V85.39, V85.41- V85.45, V85.54
Weight loss	260-263.9, 783.21, 783.22
Fluid and electrolyte disorders	276.0- 276.9

Chronic blood loss anemia	280.0, 648.20- 648.24
Deficiency anemias	280.1- 281.9, 285.21- 285.29, 285.9
Alcohol abuse	291.0- 291.3, 291.5, 291.8, 291.81, 291.82, 291.89, 291.9, 303.00- 303.93, 305.00- 305.03
Drug abuse	292.0, 292.82- 292.89, 292.9, 304.00- 304.93, 305.20- 305.93, 648.30- 648.34
Psychoses	295.00- 298.9, 299.10, 299.11
Depression	300.4, 301.12, 309.0, 309.1, 311
Hypertension, complicated and uncomplicated	401.1, 401.9, 642.00- 642.04, 401.0, 402.00- 405.99, 437.2, 642.10- 642.24, 642.70- 642.94

S1 Figure. Total Weighted Costs in 2013 US dollars Stratified by Age Category

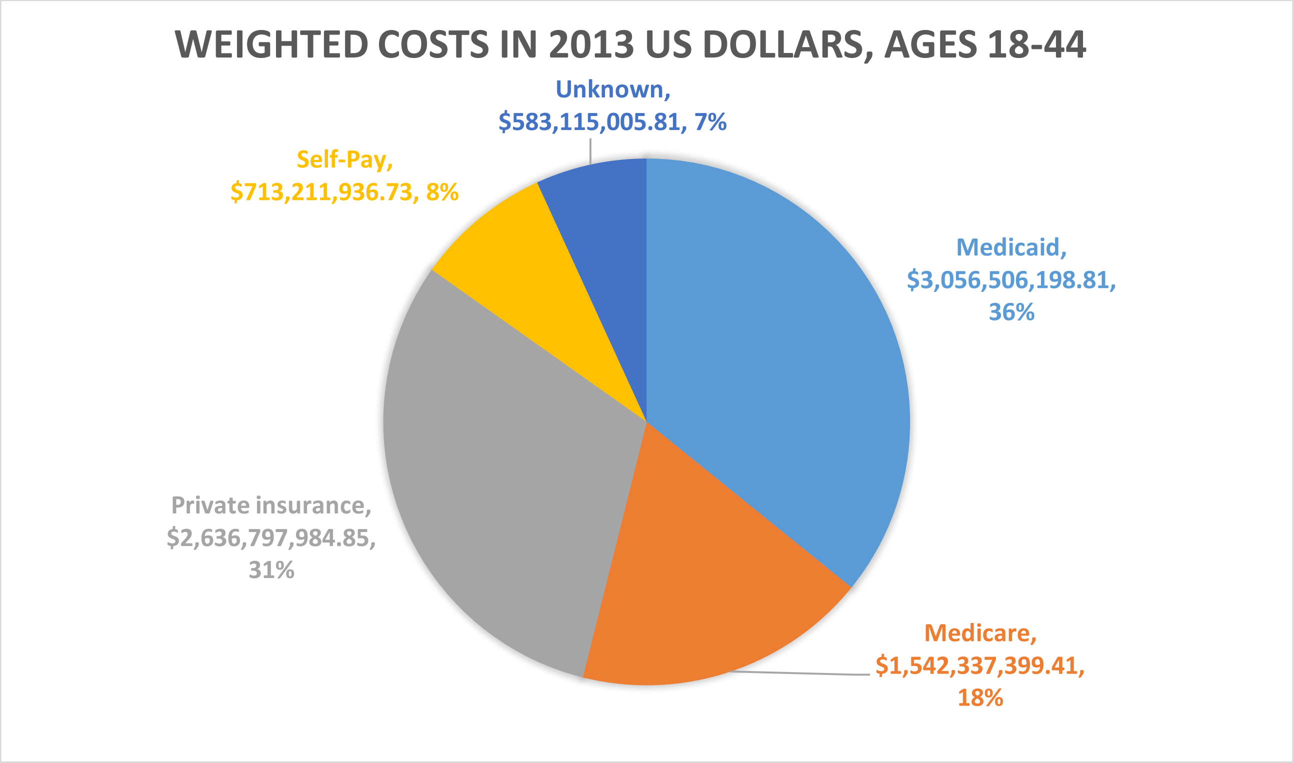

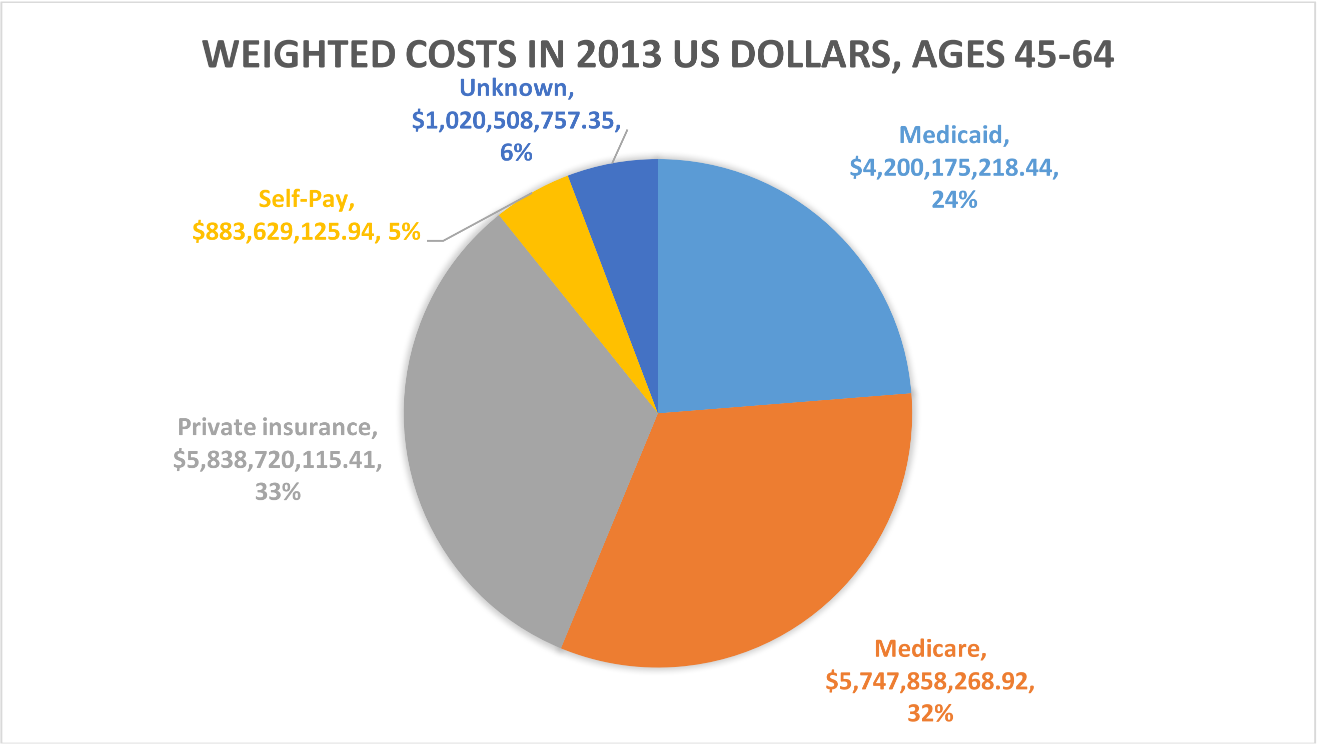

WEIGHTED COSTS IN 2013 US DOLLARS, AGES 45-64
Unknown, $1,020,508,757.35, 6%
Medicaid, $4,200,175,218.44, 24%
Self-Pay, $883,629,125.94, 5%
Private insurance, $5,838,720,115.41, 33%
Medicare, $5,747,858,268.92, 32%

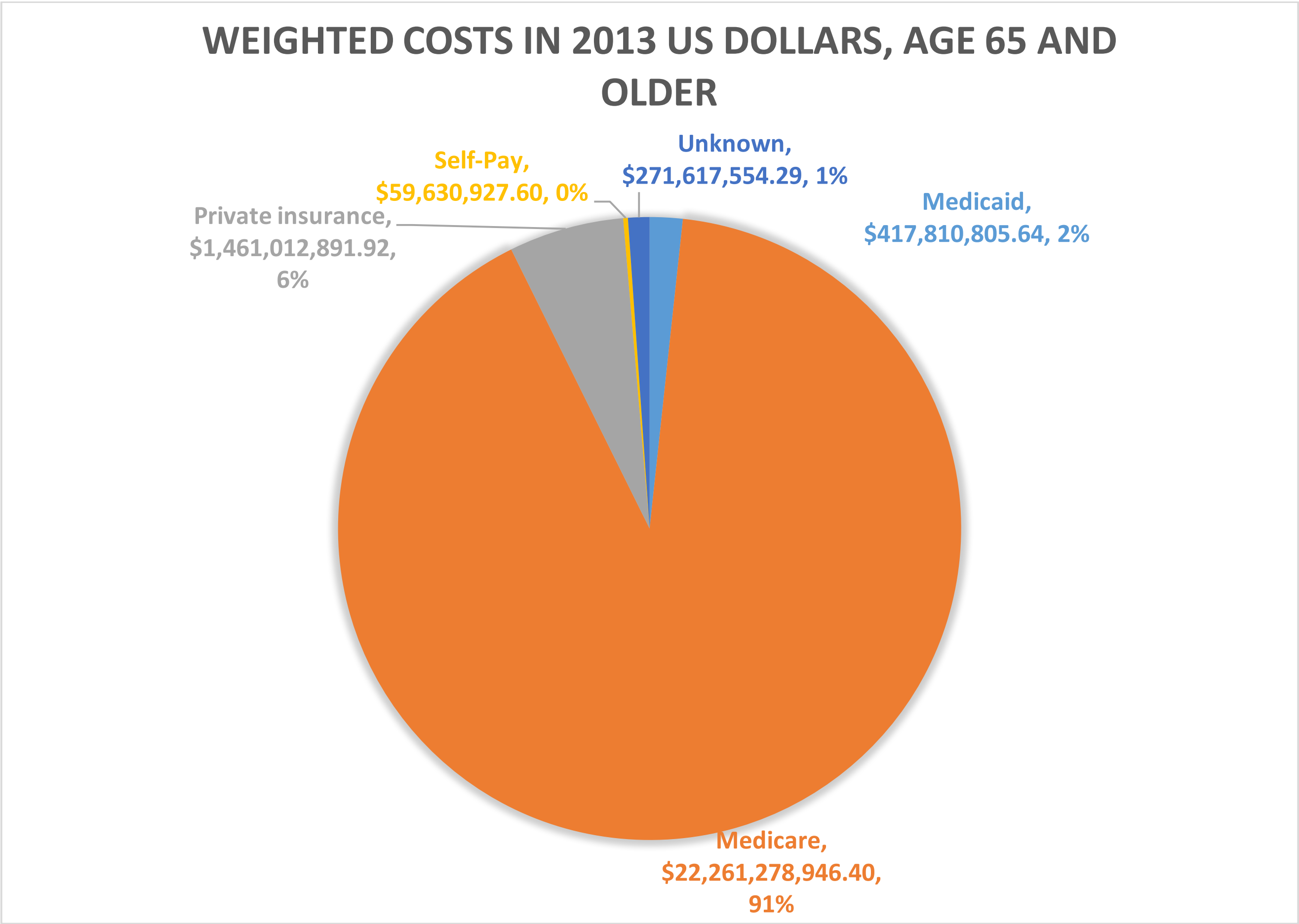
WEIGHTED COSTS IN 2013 US DOLLARS, AGE 65 AND OLDER
Unknown, $271,617,554.29, 1%
Self-Pay, $59,630,927.60, 0%
Medicaid, $417,810,805.64, 2%
Private insurance, $1,461,012,891.92, 6%
Medicare, $22,261,278,946.40, 91%

S1 Table B. Most Common Discharge Diagnoses Leading to Readmission Overall

Rank	Diagnosis	ICD-9-CM Code	Number of 30-Day Readmissions
1	Septicemia	038.XX	85,409
2	Heart Failure	428.XX	83,999
3	Cellulitis/abscess	682.XX	56,684
4	Chronic bronchitis	491.XX	50,428
5	Cardiac dysrhythmias	427.XX	49,153
6	Diabetes	250.XX	47,709
7	Pneumonia	486.XX	45,674
8	Episodic Mood Disorders	296.XX	42,947
9	Acute Kidney Failure	584.XX	40,139
10	Procedural Complications	996.XX	39,833
11	Respiratory and Chest Symptoms	786.XX	38,831
12	Schizophrenic Disorders	295.XX	33,473
13	Acute Myocardial Infarction	410.XX	31,132
14	Urinary Tract Infection	599.XX	30,186
15	Other Diseases of Lung	518.XX	28,216
16	Chronic Ischemic Heart Disease	414.XX	27,050
17	General Symptoms	780.XX	26,736
18	Instestinal Obstruction Without Hernia	560.XX	22,545
19	Diseases of Pancreas	577.XX	20,877
20	Osteoarthritis	715.XX	18,119
21	Asthma	493.XX	18,016
22	Alcohol Induced Mental Disorder	291.XX	14,682
23	Preterm or Threatened Labor	644.XX	13,844
24	Abdominal Pain	789.XX	12,806
25	Hereditary Hemolytic Anemias	282.XX	11,953
26	Conditions of Mother Complicating Pregnancy	648.XX	11,293

27	Hypertensive Kidney Disease	403.XX	11,043
28	Hypertension complicating pregnancy	642.XX	9,121
29	Acute appendicitis	540.XX	6,995
30	Other nonorganic psychoses	298.XX	6,650
31	Other Conditions of Pregnancy	646.XX	4,523
32	Abnormalities of Pelvic Organs	654.XX	4,195
33	Other care for Labor and Delivery	659.XX	3,785

S1 Table C. Discharge Diagnoses of Index Hospitalizations, Numbers, and Rates of Thirty Day Rehospitalizations

Rank	Diagnosis	ICD-9-CM Code	Number of All-Cause 30-Day Readmissions	30-Day Readmission Rate (%)	Rank	Diagnosis	ICD-9-CM Code	Number of All-Cause 30-Day Readmissions	30-Day Readmission Rate (%)
**18-44 years old**									
**Medicaid**					**Medicare**				
1	Schizophrenic Disorders	295.xx	8,599	25.1	1	Schizophrenic Disorders	295.xx	5,932	25.7
2	Episodic Mood Disorders	296.xx	8,196	18.8	2	Episodic Mood Disorders	296.xx	4,350	22
3	Preterm Labor	644.xx	7,440	21.1	3	Diabetes Mellitus	250.xx	3,807	34.8
4	Diabetes Mellitus	250.xx	6,669	30.2	4	Hereditary Hemolytic Anemias	282.xx	3,432	39.7
5	Complications of Pregnancy	648.xx	6,520	16.7	5	Complications of Surgery	996.xx	2,566	27.1
**18-44 years old**									
**Private Insurance**					**Self Pay**				
**Rank**	**Diagnosis**	**ICD-9-CM Code**	**Number of All-Cause 30-Day Readmissions**	**30-Day Readmission Rate (%)**	**Rank**	**Diagnosis**	**ICD-9-CM Code**	**Number of All-Cause 30-Day Readmissions**	**30-Day Readmission Rate (%)**
1	Preterm Labor	644.xx	5,301	18.8	1	Episodic Mood Disorders	296.xx	2,706	14.7
2	Episodic Mood Disorders	296.xx	4,568	11.3	2	Diabetes Mellitus	250.xx	2,628	19.4
3	Hypertensive Diseases of Pregnancy	642.xx	4,405	8.1	3	Cellulitis/Abscess	682.xx	1,545	10.1
4	Complications of Pregnancy	648.xx	3,616	3.9	4	Diseases of Pancreas	577.xx	1,471	16.6
5	Diabetes Mellitus	250.xx	2,911	17.3	5	Respiratory Symptoms	786.xx	1,324	25
**18-44 years old**									
**Unknown**									
**Rank**	**Diagnosis**	**ICD-9-CM Code**	**Number of All-Cause 30-Day Readmissions**	**30-Day Readmission Rate (%)**					
1	Episodic Mood Disorders	296.xx	2,706	13.8					

2	Diabetes Mellitus	250.xx	2,628	22.9
3	Cellulitis/Abscess	682.xx	1,545	9.3
4	Diseases of Pancreas	577.xx	1,471	16.9
5	Respiratory Symptoms	786.xx	1,324	21.6

45-64 years old

Medicaid					Medicare				
Rank	Diagnosis	ICD-9-CM Code	Number of All-Cause 30-Day Readmissions	30-Day Readmission Rate (%)	Rank	Diagnosis	ICD-9-CM Code	Number of All-Cause 30-Day Readmissions	30-Day Readmission Rate (%)
1	Heart Failure	428.xx	6,732	29.1	1	Septicemia	038.xx	10,158	21.8
2	Septicemia	038.xx	6,216	22.2	2	Heart Failure	428.xx	8,781	28.5
3	Chronic Bronchitis	491.xx	6,189	25.7	3	Chronic Bronchitis	491.xx	7,592	23.2
4	Schizophrenic Disorders	295.xx	5,820	26.7	4	Complications of Surgery	996.xx	7,136	22.1
5	Respiratory Symptoms	786.xx	5,149	22.2	5	Schizophrenic Disorders	295.xx	7,114	24.6

45-64 years old

Private Insurance					Self Pay				
Rank	Diagnosis	ICD-9-CM Code	Number of All-Cause 30-Day Readmissions	30-Day Readmission Rate (%)	Rank	Diagnosis	ICD-9-CM Code	Number of All-Cause 30-Day Readmissions	30-Day Readmission Rate (%)
1	Septicemia	038.xx	6,269	14.7	1	Respiratory Symptoms	786.xx	2,188	19.7
2	Cardiac dysrhythmias	427.xx	5,013	12.7	2	Heart Failure	428.xx	1,363	17.1
3	Respiratory Symptoms	786.xx	4,998	15.6	3	Alcohol induced mental disorder	291.xx	1,277	16.6
4	Complications of Surgery	996.xx	4,241	13.1	4	Diabetes Mellitus	250.xx	1,270	13.5
5	Heart Failure	428.xx	3,725	18.2	5	Episodic Mood Disorders	296.xx	1,208	15.3

45-64 years old

Unknown

Rank	Diagnosis	ICD-9-CM Code	Number of All-Cause 30-Day Readmissions	30-Day Readmission Rate (%)
1	Respiratory Symptoms	786.xx	1,522	17
2	Episodic Mood Disorders	296.xx	1,301	16.3
3	Heart Failure	428.xx	1,248	20.9
4	Septicemia	038.xx	1,179	14.1
5	Diabetes Mellitus	250.xx	1,147	16.7

65+ years old

Medicaid					Medicare				
Rank	Diagnosis	ICD-9-CM Code	Number of All-Cause 30-Day Readmissions	30-Day Readmission Rate (%)	Rank	Diagnosis	ICD-9-CM Code	Number of All-Cause 30-Day Readmissions	30-Day Readmission Rate (%)
1	Septicemia	038.xx	1,213	22.7	1	Heart Failure	428.xx	53,993	22.2
2	Heart Failure	428.xx	982	23.4	2	Septicemia	038.xx	47,860	18.4
3	Chronic Bronchitis	491.xx	504	22.9	3	Cardiac dysrhythmias	427.xx	31,648	16.9
4	Pneumonia	486.xx	467	15.9	4	Chronic Bronchitis	491.xx	29,037	20
5	Respiratory Symptoms	786.xx	451	31.9	5	Pneumonia	486.xx	27,926	16.3

65+ years old

Private Insurance					Self Pay				
Rank	Diagnosis	ICD-9-CM Code	Number of All-Cause 30-Day Readmissions	30-Day Readmission Rate (%)	Rank	Diagnosis	ICD-9-CM Code	Number of All-Cause 30-Day Readmissions	30-Day Readmission Rate (%)
1	Heart Failure	428.xx	2,514	20.3	1	Episodic Mood Disorders	296.xx	2,061	12.8
2	Septicemia	038.xx	2,364	16.7	2	Diabetes Mellitus	250.xx	1,505	21
3	Cardiac dysrhythmias	427.xx	1,841	14.6	3	Schizophrenic Disorders	295.xx	869	26.4
4	Chronic Bronchitis	491.xx	1,444	18.6	4	Diseases of Pancreas	577.xx	686	9.9
5	Pneumonia	486.xx	1,319	14	5	Cellulitis/Abscess	682.xx	648	9.9

65+ years old

Unknown

Rank	Diagnosis	ICD-9-CM Code	Number of All-Cause 30-Day Readmissions	30-Day Readmission Rate (%)
1	Heart Failure	428.xx	689	20.6
2	Septicemia	038.xx	520	16.2
3	Chronic Bronchitis	491.xx	453	20
4	Pneumonia	486.xx	380	15.8
5	Cardiac dysrhythmias	427.xx	359	15.8
